# mTORC2 controls cancer cell survival by modulating gluconeogenesis

**DOI:** 10.1038/cddiscovery.2015.16

**Published:** 2015-09-07

**Authors:** MW Khan, D Biswas, M Ghosh, S Mandloi, S Chakrabarti, P Chakrabarti

**Affiliations:** 1 Division of Cell Biology and Physiology, CSIR-Indian Institute of Chemical Biology, Kolkata, India; 2 Division of Structural Biology and Bioinformatics, CSIR-Indian Institute of Chemical Biology, Kolkata, India

## Abstract

For rapid tumor growth, cancer cells often reprogram the cellular metabolic processes to obtain enhanced anabolic precursors and energy. The molecular changes of such metabolic rewiring are far from established. Here we explored the role of mTOR (mechanistic target of rapamycin), which serves as a key regulator of cell growth, proliferation and survival, in the metabolic reprograming of cancer cells. When we inhibited mTOR in human hepatocellular carcinoma (HCC) and renal cell carcinoma (RCC) cells, using pharmacologic inhibitors or by RNA interference, we noticed shuttle of the glycolytic flux to gluconeogenesis pathway along with reduction in cellular proliferation and survival. Augmentation of gluconeogenesis was mechanistically linked to upregulation of the key gluconeogenic enzymes *PCK1* and *G6PC* expressions, enhanced lactate dehydrogenase activity and glucose-derived lipogenesis without causing any attenuation in mitochondrial function. Interestingly, concomitant knocking down of *PCK1* and not *G6PC* along with mTOR pathway could overcome the inhibition of cancer cell proliferation and survival. These observations were validated by identifying distinctive diminution of *PCK1* and *G6PC* expressions in human HCC and RCC transcriptome data. Significant correlation between mTOR-dependent upregulation of *PCK1* and cell death in different cancer cell lines further emphasizes the physiological relevance of this pathway. We reveal for the first time that inhibition of mTORC2 and consequent redistribution of glycolytic flux can have a prosurvival role in HCC and RCC cancer cells only in the presence of downregulation of gluconeogenesis pathway genes, thus identifying novel pivots of cancer cell metabolic rewiring and targets for therapy.

## Introduction

The mTOR (mechanistic target of rapamycin) kinase is considered as a critical regulator of cell size and metabolism because of its ability to couple nutrients, growth factors and oxygen availability with lysosome biogenesis and the regulation of protein and lipid synthesis.^1–3^ mTOR exists in two functionally and structurally distinct protein complexes, mTORC1 and mTORC2. mTORC1 contains raptor, as well as mLST8/G*β*L and PRAS40, whereas mTORC2 is defined by the protein rictor and also includes sin1, protor and mLST8/G*β*L.^3^ Growth factors, such as insulin and IGF, activate both the complexes via PI3K/phosphatase and tensin homolog (PTEN)/Akt signaling network.^1^

mTOR has a central role in whole-body energy metabolism by regulating both glucose and lipid homeostasis. Deregulated mTOR signaling in the mouse liver either by chronic administration of mTORC1 inhibitor rapamycin^4^ or by knocking out rictor in the liver leads to glucose intolerance, hyperglycemia, hyperinsulinemia and decreased glycogen content.^5^ These effects are mechanistically implicated to the disruption of mTORC2, which stimulates glycolysis through activation of glucokinase and inhibits gluconeogenesis by inhibiting nuclear accumulation of FoxO1 by phosphorylation of Akt at serine 473 residue.^5^ mTORC1 has also been shown to activate lipogenesis via the transcription factor sterol regulatory element-binding protein (SREBP), the master transcriptional regulator of lipogenesis.^6,7^ Mechanistically, mTORC1 activates SREBP expression, maturation and nuclear localization via separate effector pathways such as S6K1-dependent and -independent manner^6–8^ or by phosphorylating the phosphatidic acid phosphatase lipin-1 and controlling its nuclear entry.^9^ However, hyperactivation of mTORC1 in the liver by knockdown of mTORC1 inhibitor TSC1 (tuberous sclerosis 1) protects mice against diet-induced hepatic steatosis and lipogenesis^10,11^ because of feedback attenuation of Akt signaling.^11^ Similar to the TSC1-knockout mice, liver-specific rictor-knockout mice show reduced SREBP-1c activity, which could also be via an Akt-independent pathway.^12^ Thus, both mTORC1 and mTORC2 are required for SREBP-dependent hepatic lipogenesis. In addition to lipogenesis, mTORC1 was shown to suppress lipolysis in adipocyte^13^ and augment fatty acid *β*-oxidation in hepatocyte.^14,15^

As mTOR integrates both nutrient and growth factor signaling and thereby regulates cellular metabolism, growth and proliferation, hyperactivation of mTOR has been implicated to carcinogenesis. Indeed, mTOR signaling defects are often associated with cancer cell growth and survival including hepatocellular carcinoma (HCC).^16–18^ Aberrant mTOR hyperactivity has been found in 50% of patients with HCC and blocking of mTORC1 activity was shown to inhibit tumor growth in the *in vivo* mouse model.^19^ Consistent with this observation, inactivation of one negative regulator of mTOR, the PTEN, is associated with approximately half of human HCC tumors, and liver-specific PTEN-knockout mice always develop HCC at older age, suggesting a pivotal role of mTOR in hepatocellular carcinogenesis.^20^ Evidence for the direct causal role of mTOR in triggering the development of HCC was shown in liver-specific *Tsc1-*knockout mice, which develop sporadic liver cancer.^21^ However, although mTOR pathway mutations are uncommon in renal cell carcinoma (RCC),^22,23^ a highly active form of mTOR expression was found in over 60% of tumors.^24^ Thus, inhibition of hyperactivated mTOR signaling pathway has emerged as a potential target for cancer therapy. Although mTOR inhibitors rapamycin and its analogs (rapalogs), such as temsirolimus^25^ and everolimus,^26^ have been approved for the treatment of advanced RCC, the uses of these inhibitors have had only modest successes in clinical trials;^27^ however, ATP-competitive mTOR inhibitors that fully inhibit both the mTOR complexes exhibit stronger antitumor effects.^28^

One of the characteristics of cancer cells is their enhanced metabolic autonomy compared with normal cells. In the process of metabolic reprogramming, a hallmark of cancer, tumor cells take up nutrients and metabolize them in different pathways that support growth, proliferation and survival.^29,30^ For example, cancer cells convert the majority of glucose they take up into lactate even in the presence of sufficient oxygen to support oxidative phosphorylation (OXPHOS), a phenomenon known as the Warburg effect.^31^ Although much work has been carried out to understand mTOR-dependent carcinogenesis, its role in the regulation of cancer cell metabolism is still not fully understood. mTOR has been shown to be both a positive^32^ and a negative regulator of aerobic glycolysis.^33^

The focus of the present study is to elucidate the role of mTOR-mediated metabolic reprogramming and its consequences in proliferation and survival of human cancer cells. We found that blocking mTOR activity augments shuttling bulk of pyruvate into gluconeogenesis, which results in a ‘futile’ cycling of glucose that leads to halt in cancer cell proliferation and ultimately cell death.

## Results and Discussion

### Inhibition of mTOR decreases lipogenesis and enhances gluconeogenesis and LDH activity in cancer cells

**Figure 1:**
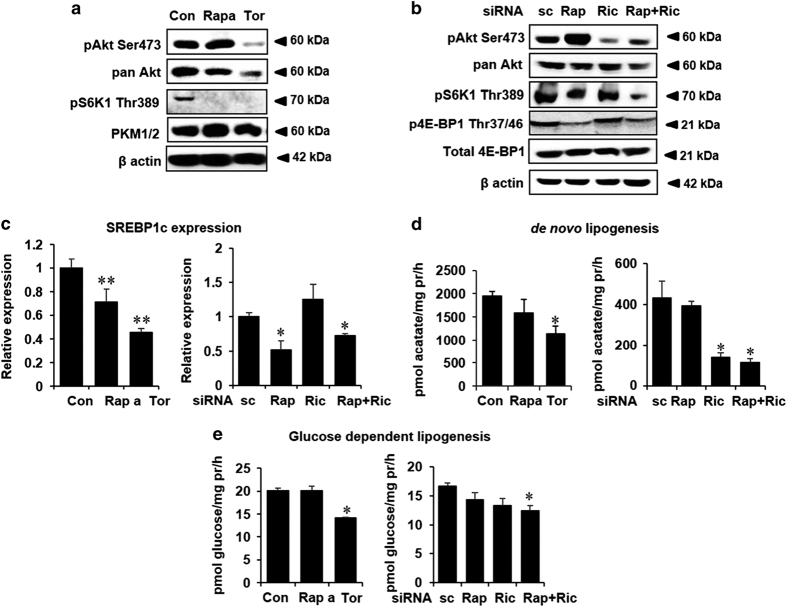
Inhibition of mTOR leads to decreased lipogenesis in human hepatoma cells. (**a**) HepG2 cells were incubated with 100 nM rapamycin (Rapa), 250 nM of torin1 (Tor) and vehicle (Con) for 24 h and whole-cell lysates were analyzed by immunoblotting with indicated antibodies. *β*-Actin serves as a loading control. (**b**) HepG2 cells were transfected with scrambled siRNA (sc) and siRNA against raptor (Rap), rictor (Ric) or both (Rap+Ric). The whole-cell lysates harvested after 48 h of transfection were analyzed by immunoblotting. (**c**) HepG2 cells were treated with Rapa and Tor for 24 h or transfected with siRNA against raptor, rictor or both as described in panels a and b. SREBP-1c mRNA levels were quantified in triplicate samples using quantitative PCR and normalized with 18S RNA expression. Data are expressed as mean±SD relative to the expression in control- (Con, left panel) or scrambled siRNA- (sc, right panel) transfected cells. (**d**) HepG2 cells were treated with Rapa or Tor (left panel) and transfected with siRNA against raptor, rictor or both (right panel), followed by incubation with ^14^C-labeled acetate for 2 h. The incorporation of ^14^C in the lipid phase was measured in triplicate samples in a *β*-scintillation counter and normalized with total cellular proteins. (**e**) Following treatment with Rapa and Tor (left panel) or transfected with indicated siRNA (right panel), HepG2 cells were incubated with ^14^C-labeled glucose for 2 h. The incorporation of ^14^C in the lipid phase was measured in as described in Materials and Methods. All panels: *n*≥3, *P<0.05, **P<0.01 compared with Con or sc cells.

To assess the role of mTORC1 and mTORC2 in controlling cancer cell metabolism, we took both pharmacological and genetic approaches for inhibiting each or both the mTOR complexes. Human HCC cells HepG2 and Huh7 were treated either with rapamycin, which specifically inhibit mTORC1, or with torin1, which binds to the ATP-binding pocket of mTOR, thereby inhibiting both the mTOR complexes. Twenty-four hour treatment with rapamycin completely blocked the phosphorylation of mTORC1 substrate S6K1 (Thr 389), whereas in addition to S6K1 phosphorylation, torin1 treatment also blocked Akt phosphorylation at serine 473, a known site phosphorylated by mTORC2 (Figure 1a and Supplementary Figure 1A). In contrast to the previous studies,^32^ neither rapamycin nor torin1 treatment altered the protein levels of pyruvate kinase isoform M (PKM1/2) in our experimental conditions (Figure 1a and Supplementary Figure 1A). To examine the specificity of the effects of the pharmacological inhibitors, HepG2 cells were transiently transfected with small interfering RNA (siRNA) targeting either raptor (mTORC1 component) or rictor (mTORC2 component) or both to inhibit mTORC1, mTORC2 and both the mTOR complexes, respectively. As shown in Figure 1b, Akt phosphorylation at serine 473 was significantly attenuated by rictor knockdown, whereas phosphorylation of S6K1 and 4E-BP1 (Thr 37/46) remained unaltered. Conversely, raptor knockdown attenuated both S6K1 and 4E-BP1 phosphorylation without affecting phosphorylation of Akt.

We next sought for the impact of mTOR blockade in cellular lipid and glucose metabolism. Both mTORC1 and mTORC2 have been implicated in the upregulation of hepatic lipogenesis partially in an SREBP-1c-dependent manner.^5,6^ As shown in Figure 1c, rapamycin modestly decreased the expression of SREBP-1c, whereas torin1 treatment and knockdown of raptor and not rictor significantly decreased SREBP-1c expression, indicating that in HepG2 cells, SREBP-1c expression is mostly regulated by mTORC1. However, the rate of *de novo* lipogenesis using ^14^C-labeled acetate was significantly decreased upon torin1 treatment (Figure 1d (left panel) and Supplementary Figure 1B) and also by rictor knockdown (Figure 1d, right panel). Taken together, our data suggest that the decrease in the rate of lipogenesis upon mTOR inhibition is not completely dependent on SREBP-1c expression levels. Interestingly, we found that the rate of lipogenesis was also significantly reduced following torin1 treatment or knockdown of both raptor and rictor when ^14^C-labeled glucose was used as tracer (Figure 1e). Thus, the conversion of glucose to lipid (Randle cycle) is at least partly modulated by mTOR.

**Figure 2:**
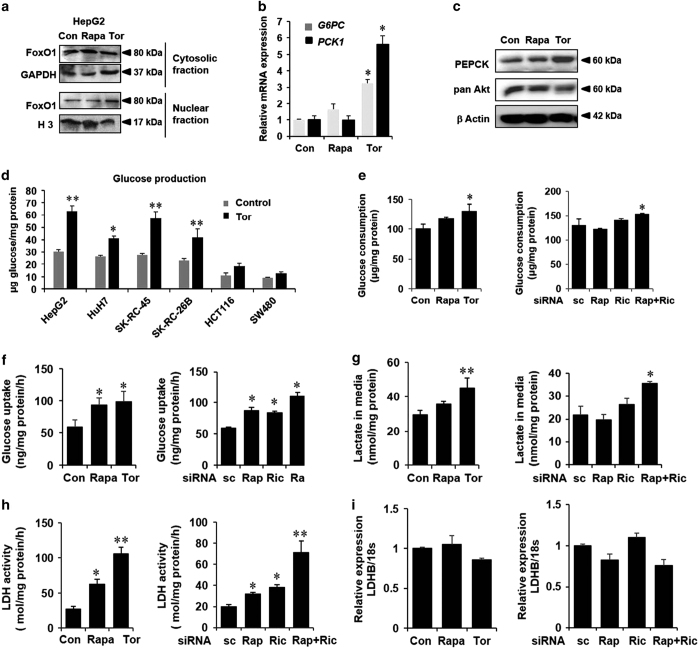
mTOR inhibition by torin1 results in the upregulation of gluconeogenesis and increased lactate production in HepG2 cells. (**a**) HepG2 cells were incubated with 100 nM rapamycin (Rapa), 250 nM of torin1 (Tor) and vehicle (Con) for 24 h and cytosolic and nuclear fractions were separated as described in the Materials and Methods, and nuclear localization of FoxO1 was analyzed by immunoblotting. GAPDH and histone H3 serve as loading controls for cytosolic and nuclear fractions, respectively. (**b**) HepG2 cells were treated with Rapa and Tor as described before and mRNA expression of *G6PC* and PCK1 were measured by quantitative PCR in triplicate samples. (**c**) Following Rapa and Tor treatment, whole-cell lysates were analyzed for PEPCK1 expression by immunoblotting. (**d**) HepG2, HuH7, SK-RC-45, SK-RC26B, HCT116 and SW480 cells were treated with Tor for 24 h, followed by incubation with glucose production media for 6 h. Glucose output in the media was measured as described in the Materials and Methods and normalized to the total cellular protein. (**e**) HepG2 cells were treated with Rapa and Tor (left panel) or transfected with siRNA against Rap, Ric or both (right panel) and glucose levels were measured in the growth media. (**f**) HepG2 cells were grown in clear bottomed black wall 96-well plates and treated with Rapa and Tor (left panel) or transfected with siRNA against Rap, Ric or both (right panel). Cells were then incubated with fluorescent 2-deoxyglucose2-NBDG for 30 min and glucose uptake was measured as described in the Materials and Methods. 2-NBDG uptake was normalized with cellular proteins. (**g**) Lactate levels were measured in the growth media after treatment with Rapa and Tor (left panel) or transfected with indicated siRNA (right panel). Lactate production was normalized with total cellular protein. (**h**) HepG2 cells were treated as indicated in panel a and LDH activity was measured in whole-cell lysates as described in the Materials and Methods. (**i**) *LDHB* mRNA expression was measured by quantitative PCR and normalized with 18S RNA expression. All panels: *n*≥3, *P<0.05, **P<0.01 compared with Con or sc cells.

As lipogenesis is coupled to glucose metabolism^34^ and mTOR has been shown to regulate hepatic glycolysis and gluconeogenesis, we next examined the effects of mTOR inhibition on glucose metabolism. Inhibition of mTORC2 leads to decreased Akt phosphorylation, which would induce nuclear translocation of FoxO1 and the upregulation of FoxO1 target gluconeogenic genes such as *G6PC* and *PCK1*. Thus, gluconeogenesis is upregulated in mice with liver-specific knockdown of rictor.^5^ As expected, nuclear FoxO1 content was increased with a concomitant decrease in cytosolic FoxO1 in HepG2 cells treated with torin1 but not with rapamycin (Figure 2a). Expression of *G6PC* and *PCK1* genes and phosphoenolpyruvate carboxykinase (PEPCK1) protein levels were increased upon torin1/rictor knockdown (Figures 2b and c and Supplementary Figures 2A and B) and MK-2206 (pan-Akt inhibitor) treatment (Supplementary Figures 2C and E). As glycogen synthase kinase 3 (GSK3) is also a well-characterized downstream target of Akt, we asked whether GSK3 is the main effector for mTORC2-dependent increased gluconeogenic gene expression. To this effect, we treated HepG2 cells with 30 *μ*M of GSK3 inhibitor SB-415286 in the presence and absence of torin1. We found that GSK3 inhibition alone did not alter PEPCK protein and *PCK1* expression (Supplementary Figures 2D and F). The rate of gluconeogenesis as measured by glucose production was also significantly elevated following treatment with torin1 in HCC and RCC but not in CC cells (Figure 2d). MK-2206 treatment could also enhance glucose production in HepG2 cells, whereas treatment with SB-415286 showed no significant change (Supplementary Figures 2G and H). As glucose production was enhanced when mTOR is inhibited, it was expected that cells would consume less glucose in similar experimental conditions. However, we did not find any drop in cellular glucose consumption as assayed by glucose concentrations in the media when mTOR was inhibited either by torin1 treatment or siRNA-mediated knockdown of raptor and rictor (Figure 2e and Supplementary Figure 1C). Indeed, glucose concentrations in the media showed an increasing trend in our experimental conditions. Cellular glucose uptake (Figure 2f and Supplementary Figure 1D) and secretion of lactate in the media (Figure 2g and Supplementary Figure 1E) were also significantly upregulated following inhibition of mTOR.

To investigate the mechanism of increased lactate production, we measured cellular lactate dehydrogenase (LDH) activity. LDH activity was found to be significantly enhanced upon mTOR inhibition (Figure 2h). LDH is a tetrameric enzyme comprising of isoform A and/or B, of which LDHB is the major isoform in the liver. It catalyzes the reversible reaction from pyruvate to lactate and its upregulation has been implicated in various cancers. Furthermore, LDH activity has been shown to be crucial for cancer cell survival.^35^ Although LDHB expression is under the control of hyperactivated mTOR in mouse embryonic fibroblasts, mTOR-dependent *LDHB* expression in HepG2 cells may not be critical.^36^ Consistent with this observation, we could not detect any significant change in the *LDHB* expression upon inhibition of mTOR (Figure 2i). Thus, the metabolic alteration caused by mTOR inhibition is probably somewhat compensated by the increased LDH activity and cell survival.

### Inhibition of mTOR diminishes glycolytic flux to mitochondria

**Figure 3:**
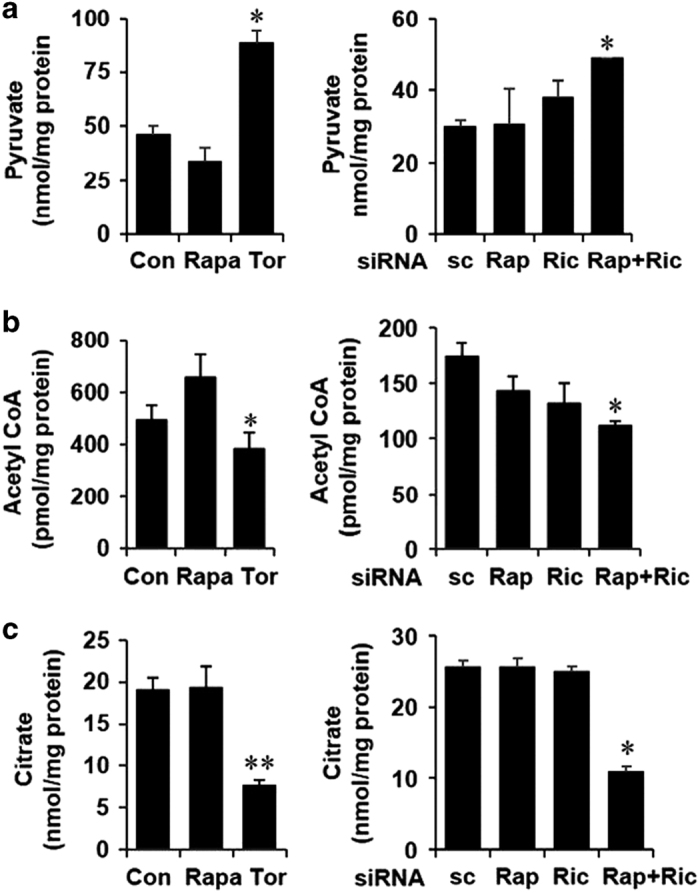
mTOR inhibition reduces metabolic flux to the mitochondria. (**a**–**c**) Cells were treated/transfected as described earlier and pyruvate, acetyl-coA and citrate levels were measured in triplicate samples in whole-cell lysates and normalized to the total cellular protein as described in Materials and Methods. All panels: *n*=4, *P<0.05, **P<0.01 compared with Con or sc cells.

In the glycolytic pathway, glucose is metabolized to form acetyl-CoA, which condensed with oxaloacetate eventually generates citrate in the mitochondrial TCA cycle. The cytosolic pool of acetyl-CoA, the precursor of fatty acid synthesis, is predominantly contributed by the hydrolysis of mitochondria-derived citrate by the cytosolic ATP citrate lyase.^37^ Our results showing a decrease in the *de novo* lipogenesis as well as in the glucose-dependent lipogenesis could be attributed to the downregulation of SREBP-1c expression or because of a decrease in the available metabolic flux or both (Figures 1c–e). To examine whether mTOR modulates the metabolic flux in the glycolysis and TCA cycle, we measured concentrations of pyruvate, acetyl-CoA and citrate in mTOR-inhibited cells. Cellular pyruvate levels were found to be significantly enhanced (Figure 3a), whereas cellular acetyl-CoA and citrate levels were significantly reduced by torin1 treatment as well as by knockdown of raptor and rictor (Figures 3b and c).

Taken together, our data suggest that in addition to the downregulation of SREBP-1c expression, inhibition of mTOR also increases cellular pyruvate levels while decreasing cellular citrate, thereby creating a metabolic state where glycolytic flux to the mitochondria or the conversion of pyruvate to acetyl-CoA is attenuated. Moreover, our results indicate that blocking mTOR activity shuttles the glycolytic flux to lactate and to gluconeogenesis. Inhibition of mTOR thereby leads to a ‘futile’ glucose metabolic cycle where both gluconeogenesis and glucose consumption are upregulated. We thus assume that following mTOR blockade, such an unfavorable rewiring of glucose utilization in cancer cells can detrimentally affect cellular growth and survival.

### Mitochondrial function remains unaltered following mTOR inhibition

**Figure 4:**
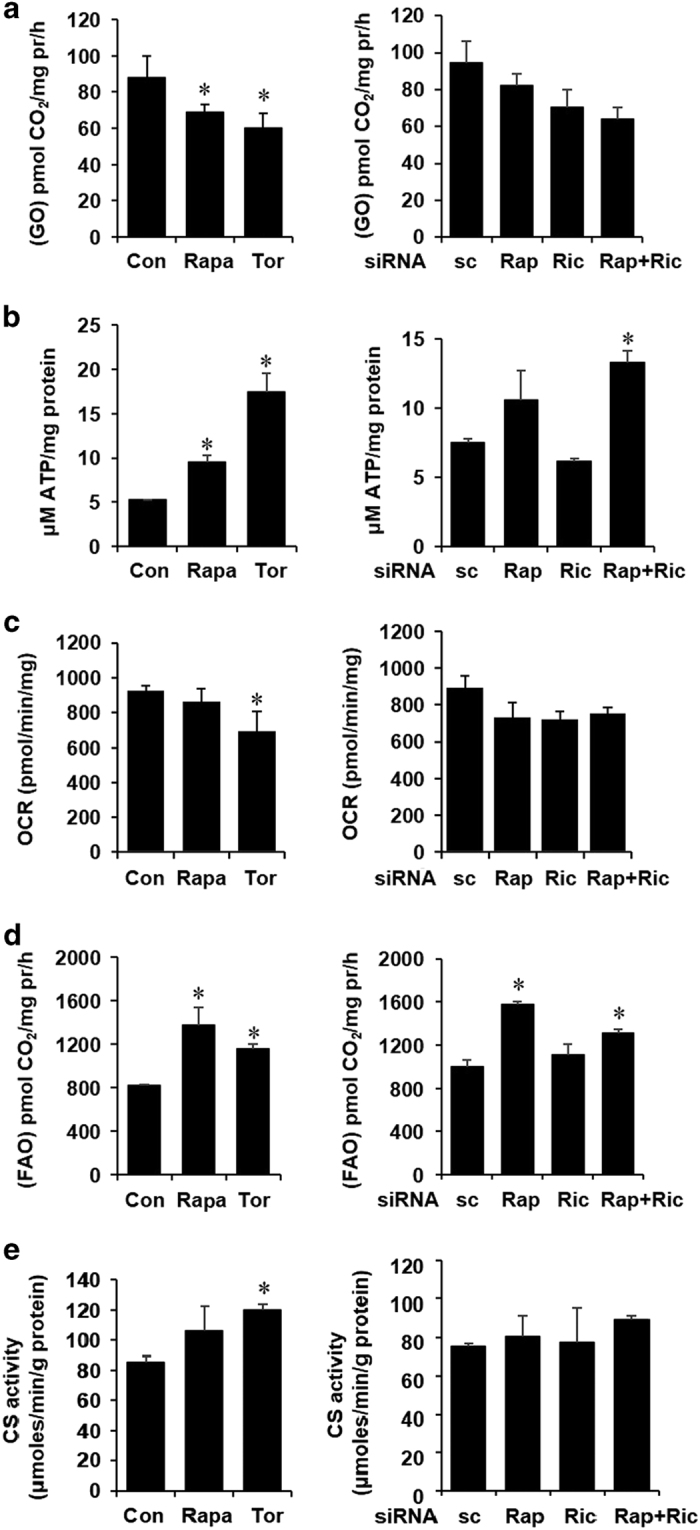
mTOR inhibition does not alter mitochondrial function. (**a**) HepG2 cells were treated with Rapa and Tor (left panel) or transfected with siRNA against Rap, Ric or both (right panel). Cells were then incubated with ^14^C-labeled glucose for 2 h and released^ 14^C-labeled CO_2_ was trapped in the filter paper and counted as described in the Materials and Methods. Complete oxidation of glucose (GO) was normalized to cellular protein. (**b**) Cells were treated as described before and ATP levels were estimated in whole-cell lysates. (**c**) OCR was measured in the presence of 25 mM glucose as indicated. (**d**) HepG2 cells were treated as described before and incubated with ^14^C-labeled palmitic acid for 2 h. Fatty acid oxidation (FAO) was quantified as described in the Materials and Methods. (**e**) Citrate synthase (CS) activity was measured in the mitochondrial fraction of HepG2 cells treated as indicated. All panels: *n*≥3, *P<0.05, **P<0.01 compared with Con or sc cells.

As inhibition of mTOR induces shuttling of glucose into lactate and the gluconeogenic pathway, we thought that complete mitochondrial glucose oxidation should simultaneously be attenuated. Consistent with previous results, as shown in Figure 4a, ^14^CO_2_ generated from complete oxidation of radiolabeled glucose was significantly reduced upon mTOR inhibition. However, cellular ATP concentrations were increased under similar experimental conditions (Figure 4b). To assess the state of mitochondrial respiration following inhibition of mTOR, we measured cellular oxygen consumption rate (OCR). However, although we could not detect any difference in the basal OCR when cells were incubated in 5 mM glucose (not shown), under high glucose conditions (25 mM), OCR was reduced in torin1-treated cells (Figure 4c). Taken together, our data indicate that inhibition of mTOR does not significantly alter OXPHOS in HepG2 cells. To elucidate the possible mechanism by which mTOR-inhibited cells maintain OXPHOS, we measured the rate of fatty acid oxidation (FAO) and found that FAO was significantly increased in cells treated with rapamycin and torin1. Consistent with this result, FAO was upregulated when raptor or both raptor and rictor were knocked down (Figure 4d). To further biochemically examine whether the inhibition of mTOR causes any change in the mitochondrial content and oxidative capacity, CS activity was measured. As shown in Figure 4e, neither pharmacological inhibition nor siRNA-mediated knockdown of mTOR components led to any significant change in CS activity.

Collectively, these observations indicate that inhibition of mTOR leads to an attenuation of complete oxidation of glucose because of shuttling of the glucose-derived metabolites into lactate and into the gluconeogenic pathway. This metabolic reprogramming results in the lowered supply of cellular acetyl-CoA leading to a decreased rate of lipogenesis. However, mitochondrial OXPHOS remains unaltered putatively because of concomitant increase in the rate of FAO.

### Knockdown of PCK1 in mTOR-inhibited cells improves cellular survival

**Figure 5:**
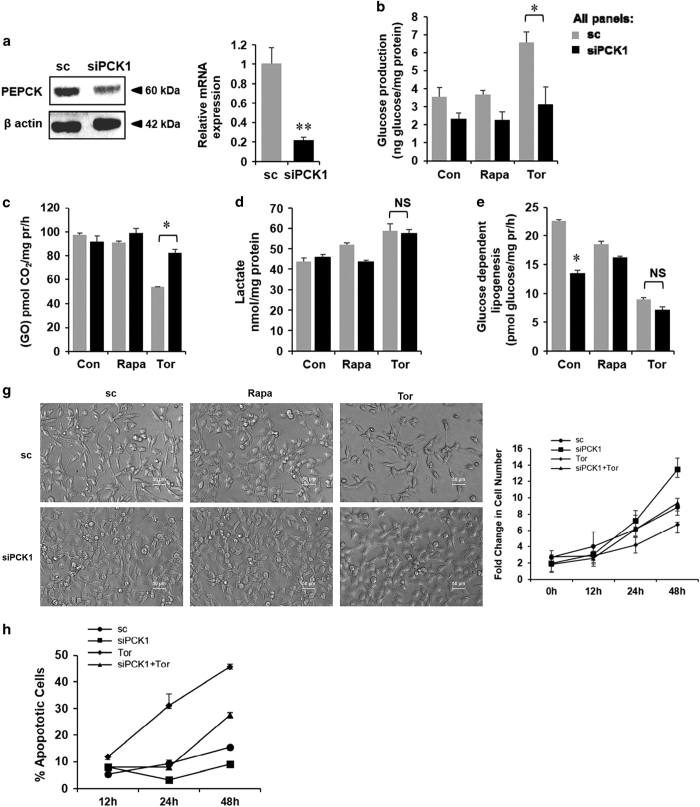
PCK1 knockdown augments survival and proliferation in mTOR-inhibited cells. (**a**) HepG2 cells were transfected with scrambled siRNA (sc) and siRNA against *PCK1*. Whole-cell lysates were analyzed for PEPCK protein expression (left panel). mRNA levels of PCK1 were analyzed by quantitative PCR and normalized to 18S RNA expression (right panel). (**b**) After 24 hposttransfection with scrambled (sc) and siPCK1, cells were treated with Rapa and Tor for another 24 h as indicated and glucose production was measured in the media. (**c**) Oxidation of ^14^C-labeled glucose measured as described in Materials and Methods after siPCK1 knockdown followed by Rapa and Tor treatment for 24 h. (**d**) Lactate production in the media was measured after siPCK1 knockdown followed by Rapa and Tor treatment. (**e**) Partitioning of ^14^C-labeled glucose in the lipid phase was measured after siPCK1 knockdown, followed by Rapa and Tor treatment. (**f**) Cell proliferation using MTT assay after siPCK1 knockdown followed by Rapa and Tor treatment. (**g**) Phase-contrast micrograph of HepG2 cells with Rapa and Tor treatment with or without *PCK1* depletion (left panel). FC in cell number following siPCK1 transfection and Tor treatment up to 48 h (right panel). (**h**) Percentage of early and late apoptotic cells were determined after treatment siRNA transfection and treatment with Tor (250 nM) by flow cytometry after the stipulated time periods as indicated using Annexin V and PI staining as described in Material and Methods. All panels: *n*≥3, *P<0.05, **P<0.01 compared with Con or sc cells.

To examine whether metabolic reprogramming caused by mTOR inhibition can modulate cellular physiology, we assayed for HepG2 cell proliferation and survival following rapamycin and torin1 treatment. Torin1 treatment caused a significant decrease in cellular proliferation, while rapamycin had no or minimal effect (Supplementary Figures 3A and B). Similarly, both MK-2206 and SB-415286 caused a significant decrease in cellular proliferation (Supplementary Figures 3C and D). Cell death, assayed by phosphatidyl serine exposure to the outer leaflet of the cell membrane, was increased by both rapamycin and torin1, the latter being more cytotoxic (Supplementary Figures 3E and F). However, neither MK-2206 nor SB-415286 could further augment torin1-induced cell death (Supplementary Figures 3G and H). As lactate serves as one of the gluconeogenic precursors and inhibition of mTOR is associated with a parallel increase in glucose production (Figure 2d), we sought to investigate the role of gluconeogenesis pathway in cellular proliferation and survival. To this end, using siRNA we knocked down *PCK1*, which catalyzes the first committed step of gluconeogenic pathway, and *G6Paseα* (catalytic subunit), which catalyzes the last step in the gluconeogenesis pathway. As shown in Figure 5a (Supplementary Figure 4A), transfection of siRNA reduced *PCK1* gene and protein expressions by 80% and 50%, respectively. As expected, knockdown of *PCK1* significantly decreased the rate of glucose production in torin1-treated cells (Figure 5b) with a concomitant increase in glucose oxidation (Figure 5c). Interestingly, depletion of *PCK1* did not alter lactate production (Figure 5d) or the incorporation of glucose into the cellular lipid pool (Figure 5e). In both HepG2 (HCC) and SK-RC-45 (RCC) cells, proliferation showed an increasing trend following knockdown of *PCK1* (Figure 5f and Supplementary Figure 4B), and in contrast, depletion of *PCK1* significantly reduced both the early and late stage of cell death over 48 h following torin1 treatment (Figures 5g and h and Supplementary Figures 5A and D). Although decrease in glucose production by knocking down *G6Paseα* did not reach statistical significance (Supplementary Figures 6A and B), si*G6Paseα* transfection resulted in a decrease in cellular proliferation and survival. However, depletion of *G6Paseα* did not affect the cytotoxic response to torin1 (Supplementary Figures 6C and D), indicating that *G6Paseα* may not be an important regulator of cell survival in the context of mTOR inhibition. Taken together, our data indicate that torin1-dependent upregulation of *PCK1* (Figure 2b) shuttles the glycolytic flux into the gluconeogenic pathway, which is associated with enhanced cell death. As mTOR functions as a critical regulator of cellular energy balance, perturbation of glucose metabolism by torin1 treatment could induce a metabolic stress, which is not compatible with the survival of rapidly dividing cells.

### PCK1 expression is associated with cancer cell survival

**Figure 6:**
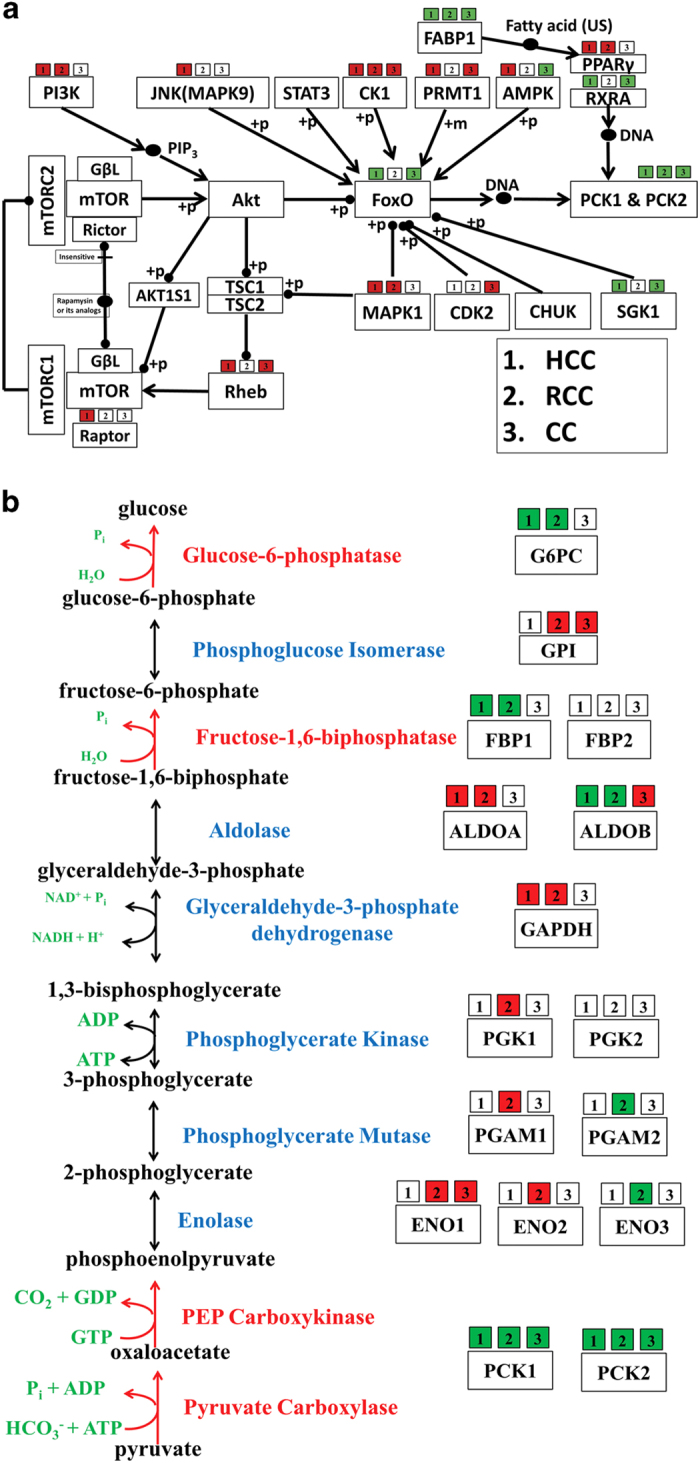

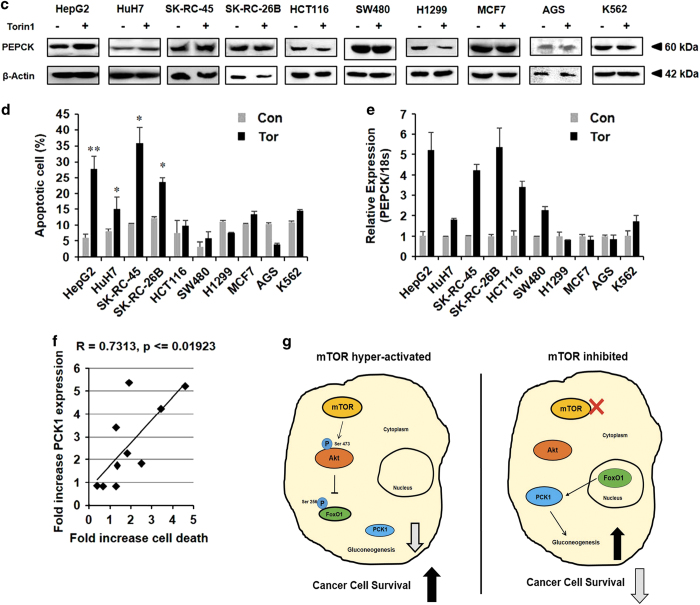
mTOR-dependent PCK1 expression is associated with cancer cell survival. (**a**) mTOR-PCK1 subnetwork constructed from the KEGG pathways. Network consists of node as protein/gene (or complex) and edges are inhibition or activation connection. Differential expression information for (1) HCC, (2) RCC and (3) CC is provided above each node. Red represents upregulation, green represents downregulation and white represents no significant change or not found in this analysis. (**b**) Systematic representation gluconeogenesis pathway. Enzymes are marked in blue color and differential expression information for (1) HCC, (2) RCC and (3) CC is provided above each gene. Red represents upregulation, green represents downregulation and white represents no significant change or not found in this analysis. Reaction colored in red are rate-limiting steps in the pathway. (**c**) Different cancer cell lines, HCC (HepG2, HuH7), RCC (SK-RC-45, SK-RC-26B), CC (HCT116, SW480), lung carcinoma (H1299), breast cancer (MCF7), gastric adenocarcinoma (AGS) and leukemia (K562), were treated with torin1 for 24 h and the cell lysates were analyzed by immunoblotting for PEPCK. *β*-Actin serves as a loading control. (**d**) Cell lines were treated with torin1 for 24 h and total RNA was extracted for determining the expression levels of PCK1. (**e**) All cell lines were treated with torin1 for 24 h and apoptosis induction was measured using Annexin V/PI by flow cytometry as described in Materials and Methods. (**f**) Correlation plot for fold increase in PCK1 RNA expression and fold increase in cell death upon torin1 treatment. (**g**) Schematic representation of hypothesis. Cancer cells downregulate gluconeogenic program to use glucose via the shunt pathways. mTOR blockade induces nuclear localization of FoxO leading to the upregulation of PCK1 and inhibition in cancer cell proliferation and survival. All panels: *n*≥3, *P<0.05, **P<0.01 compared with Con cells.

As liver and renal cortex are the two most important sites of gluconeogenesis in human, malignant tumors developed from these tissues would possibly be more sensitive to mTOR-dependent enhancement of gluconeogenesis as metabolic insult and cell death. Conversely, downregulation of key gluconeogenic enzymes including PEPCK1 would be a survival mechanism for these tumor cells. To examine whether *PCK1* expression is altered in human cancers (e.g., HCC, RCC and CC), we analyzed differential expression patterns of genes involved in the mTOR-PCK1 metapathway subnetwork (see Materials and Methods). *PCK1* was seen to be significantly downregulated (raw values for all data sets in this study are available at www.hpppi.iicb.res.in/images/oncomine_pck1.zip) in all these three cancers (Figure 6a). FOXO, which is a positive regulator of *PCK1* gene, is observed to be downregulated in HCC and CC patient samples without any change in AKT and mTOR expressions. However, raptor, which is a key component of mTORC1 complex, was found to be upregulated, whereas other key genes such as *Rheb* and *PI3K*, which directly or indirectly impact mTOR and AKT, were also overexpressed. Hence, it could be postulated that the expressions of mTOR complex and AKT are sufficiently high to downregulate FOXO, which in turn may negatively control *PCK1* expression. Next, we checked the expression levels of other gluconeogenesis enzymes in HCC, RCC and CC tumors (Figure 6b). Interestingly, all the rate-limiting enzymes including *PCK1*, *FBP1* and *G6PC* are found to be downregulated in HCC and RCC tumors compared with the adjacent tumor-free tissues, suggesting suppression of gluconeogenesis pathway, which putatively endows a survival cue to these tumors.

As a proof of concept, we analyzed 10 different cancer cell lines for torin1-dependent *PCK1* expression and cell survival. We found that torin1 treatment resulted in the upregulation of *PCK1* gene expression only in HCC, RCC and CC cells, whereas PEPCK1 protein was significantly increased only in HCC and RCC cells (Figures 6c and d). Concomitant with the increase in PEPCK protein levels, we found significant cell death in HCC and RCC cells (Figure 6e). Further, we found a significant positive correlation between the torin1-dependent upregulation of *PCK1* and cell death (Figure 6f). Notably, in CC cell lines torin1-dependent increase in *PCK1* gene expression did not result in increased protein expression (Figures 6c–e) and glucose production (Figure 2d), which may explain the ineffectiveness of torin1 as a death-inducing agent in these cells. Taken together, our results suggest that in HCC and RCC cells repartitioning of glucose for the optimum cell proliferation and survival is associated with suppression of the gluconeogenic pathway and mTORC2 inhibition leads to cell death by upregulating it.

The metabolic program of various cancer cells characterized by increased glucose consumption associated with elevated rate of lactate production in normoxia or aerobic glycolysis is known as the Warburg effect. The role of oncogenes in activating glucose uptake, glycolysis and blocking the entry of glucose carbons to the mitochondria have been well documented.^38–41^ However, little attention has been focused on gluconeogenesis, which is potentially poised to have an important role in the switch to aerobic glycolysis in tumor cells. Bhalla *et al.* ^42^ showed that in hepatocytes oncogenic cyclin D1 inhibits gluconeogenesis via the inactivation of peroxisome proliferator-activated receptor *γ* coactivator 1*α* (PGC1*α*), which stimulates the expression of gluconeogenic genes *PCK1* and *G6PC*. Notably, PGC1*α* expression has been shown to be reduced in different cancers, including HCC. Conversely, the tumor suppressor p53 was shown to promote the expression of key gluconeogenic mediators *PCK1* and *G6PC* and thereby enhance hepatic glucose production.^43^ Role of gluconeogenesis in carcinogenesis was further appreciated when loss of fructose-1,6-bisphosphatase (FBP1), a critical regulator of gluconeogenesis, was shown to be essential for the induction of epithelial-to-mesenchymal transition in human breast cancer.^44^ Interestingly, loss of FBP1 expression because of promoter DNA methylation has been observed before in the liver, colon and gastric cancers^45,46^ and depletion of FBP1 in RCC tumors was found to be correlated with advanced tumor stage and worse patient prognosis.^47^ Notably, steroid-induced enhancement in gluconeogenesis reduced the formation of liver tumors in a mouse model of HCC.^48^ In line with these observations, our data indicate that augmenting gluconeogenesis by mTORC2 blockade could also deleteriously affect HCC and RCC cancer cell proliferation and survival.

Consistent with the notion that upregulation of gluconeogenesis can be detrimental to the cancer cell survival, our results suggest that upregulation of gluconeogenic pathway following mTORC2 inhibition creates a metabolic stress where bulk of the glucose is exported outside the cells. Such futile metabolic rewiring is partly responsible for torin1-induced cell death as coinhibition of *PCK1* significantly increased cell survival (Figure 6g). Conversely, in cell lines where mTOR inhibition could not lead to the upregulation of gluconeogenesis did not undergo significant cell death. Our findings thus uncover an important role of mTORC2 in cancer biology and possibly explain the phenomenon as to why mTOR inhibitors are effective in certain cancer types only.

## Materials and Methods

### Materials and antibodies

Reagents were obtained from the following sources: torin1 from Tocris Biosciences (Bristol, UK); rapamycin from Calbiochem; MK-2206 from Sigma (St. Louis, MO, USA); SB-415286 from Santa Cruz Biotechnology Inc. (Dallas, TX, USA); Dulbecco’s modified Eagle’s medium (DMEM), fetal bovine serum (FBS), trypsin, penicillin/streptomycin and Lipofectamine 2000 transfection reagent from Life Technologies (Carlsbad, CA, USA); the complete protease and phosphatase inhibitor mixture from Roche Applied Science (Mannheim, Germany); the antibodies to pan-Akt, phospho-Ser-473 Akt, phospho-Thr-389 S6K1 and phospho-Thr-37/46 4E-BP1 from Cell Signaling Technologies (Danvers, MA, USA); the antibodies to total 4E-BP1, total S6K1 and PEPCK from Santa Cruz Biotechnology Inc.; the PKM1/2 antibody from Thermo (Waltham, MA, USA); and the *β*-actin antibody from Sigma. siRNA against Raptor, Rictor, PCK1, G6Pase*α* and scramble siRNA were obtained from Santa Cruz Biotechnology, Inc. All other reagents were from Sigma.

### Cell culture

HepG2 and HuH7 (HCC), HCT116 and SW480 (colorectal carcinoma, CC), MCF7 (breast adenocarcinoma) cell lines were cultured in DMEM with 10% FBS and penicillin/streptomycin. SK-RC45, SK-RC26B (RCC) cell lines were cultured in RPMI with 20% FBS and penicillin/streptomycin. H1299 (non-small-cell lung carcinoma) and K562 (chronic myelogenous leukemia) cell lines were cultured in RPMI with 10% FBS and penicillin/streptomycin. AGS (gastric adenocarcinoma) cell line was maintained in F-12K medium with 10% FBS and penicillin/streptomycin. All the cell lines were maintained at 37 °C in 5% CO_2_. The cells were cultured at a density that allowed cell division throughout the course of the experiment. Transient transfections with siRNA against raptor/rictor/PCK1/G6PC*α* were performed using Lipofectamine 2000 transfection reagent according to the manufacturer’s instructions.

### Cell lysis and immunoblotting

Cells were rinsed with ice-cold PBS before lysis in buffer containing 50 mM Tris-HCl (pH 7.4), 100 mM NaCl, 1 mM EDTA, 1 mM EGTA and 1% Triton X-100 with protease/phosphatase inhibitor mixture. The soluble fractions of cell lysates were isolated by centrifugation at 15 000 r.c.f. for 15 min at 4 °C. Samples of the cellular lysates containing an equal amount of proteins were resolved by SDS-PAGE and transferred to PVDF membrane (Millipore, Billierica, MA, USA). Proteins were then visualized with enhanced chemiluminescence using Luminata Classico Western HRP substrate (Millipore).

### Glucose uptake

Glucose uptake experiments were performed using 2-NBDG (2-(*N*-(7-nitrobenz-2-oxa-1,3-diazol-4-yl)amino)-2-deoxyglucose) (Invitrogen, Carlsbad, CA, USA) according to the manufacturer’s protocol. Briefly, cells were plated in a 96-well black clear bottom plate (Brand, Wertheim, Germany). After treatment, cells were washed three times with warm 1× PBS and incubated for 30 min in zero glucose DMEM containing 75 *μ*M 2-NBDG. Cells were then washed with cold PBS three times. To each well, 200 *μ*l of PBS was added and the relative fluorescence was measured in a fluorimeter (Synergy H1 multimode microplate reader; Biotek (Winooski, VT, USA); excitation 485 nm, emission 535 nm). The assay was normalized by the total cellular protein.

### CS assay

CS activity was measured by the method of Spinazzi *et al.* ^49^ Briefly, cells were treated with rapamycin (100 nM)/torin1 (250 nM) for 24 h and the mitochondrial fractions were separated. To a 96-well microplate, 100 *μ*l of Tris (200 mM, pH 8.0) with 0.2% Triton X-100, 20 *μ*l of 1 mM DTNB, 12.8 *μ*l of 4.7 mM acetyl-CoA and 50 *μ*g of protein sample was added. The volume was adjusted to 200 *μ*l with distilled water. The reaction was started by adding 10 *μ*l of 10 mM oxaloacetic acid, and the increase in absorbance was monitored at 412 nm for 3 min. Activity was expressed as nmol DTNB reduced per min per mg protein (*ε*412=13.6/mmol/l/cm).

### Glucose production assay

Glucose production was measured by the method of Yoon *et al*.^50^ with minor modifications. Cells were treated and kept in growth medium for 24 h and then washed with glucose output media (118 mM NaCl, 4.7 mM KCl, 1.2 mM MgSO4, 1.2 mM KH_2_PO_4_,1.2 mM CaCl_2_, 20 mM NaCO_3_, 25 mM HEPES (pH 7.4) and 0.025% BSA) and the glucose output was measured in fresh glucose output media supplemented with gluconeogenic substrates (20 mM lactate, 2 mM pyruvate, 10 mM glutamine) for 6 h. Glucose levels in the media were measured by glucose assay reagent (Sigma-Aldrich, St. Louis, MO, USA) and normalized to total protein.

### Oxidation studies

^14^C-labeled substrate oxidation to ^14^CO_2_ was performed as described earlier^51^ with minor modifications. Cells at ~80–90% confluency were treated with rapamycin (100 nM)/torin1 (250 nM) for 24 h. Cells were then serum starved for 2 h and 0.5 *μ*Ci of radiolabeled 1-^14^C palmitate (BRIT, Mumbai, India) or 0.5 *μ*Ci of [^14^C(U)]d-glucose (Perkin-Elmer, Waltham, MA, USA) was added to 1.5 ml of media. A Whatman chromatography paper cut to the size of a 6-well plate was soaked in 3 M NaOH and placed on the lid of the plate, such that each well is covered. The cells were incubated at 37 °C for 2 h. Following the incubation, 500 *μ*l of 70% perchloric acid was added to each well and the released CO_2_ was trapped in the Whatman paper for 1 h. The filter paper was left for drying overnight and the ^14^C levels were estimated using a *β*-counter (Perkin-Elmer). Cells were scrapped and pelleted down after a brief centrifugation at 5000 r.c.f for 5 min at 4  °C. The pellet was washed two times with 1× PBS and lysed using 0.5 N NaOH, vortexed and centrifuged to isolate the cell lysate at 18 000 r.c.f. for 10 min at 4 °C. Experiments were carried out in triplicate and normalized to total cellular protein.

### Lipogenesis

Incorporation of radiolabeled acetate or glucose in cells was carried out following the method of Chakrabarti *et al.* ^13^ Briefly, cells at ~80–90% confluency were treated with rapamycin (100 nM)/torin1 (250 nM) for 24 h. After the stipulated time period, cells were serum starved for 2 h followed by incubation with either 1 *μ*Ci of 1,2-^14^C-sodium acetate (BRIT) or 0.5 *μ*Ci of [^14^C(U)]d-glucose for 2 h and total intracellular lipids were extracted with hexane and 2-propanol (3:2 (vol/vol)) mixture. Incorporation of [1,2-^14^C]acetate or [^14^C(U)]d-glucose into the lipid phase was assayed by scintillation counting. All the experiments were carried out in triplicate and normalized by protein concentration in samples.

### Cell fractionation

Cytosolic, mitochondrial and nuclear fractions were separated by following the method of Holden and Horton.^52^ Briefly, cells at ~80–90% confluency harvested by adding 1× trypsin and washed two times with ice-cold 1× PBS. The pellet was resuspended in 400 *μ*l of ice-cold buffer 1 (150 mM NaCl, 50 mM HEPES (pH 7.4), 25 *μ*g/ml digitonin, protease inhibitor cocktail). This was further incubated at 4 °C in end-over-end rotation for 10 min, following which it was centrifuged at 2000 r.c.f. to pellet the cells. The supernatant was aspirated to obtain the cytosolic fraction. The cells were further washed with ice-cold 1× PBS and centrifuged at 100 r.c.f. at 4 °C to remove any digitonin. The pellet was resuspended by vortexing in 400 *μ*l of ice-cold buffer 2 (150 mM NaCl, 50 mM HEPES, pH 7.4, 1% NP40, protease inhibitor) and incubated on ice for 30 min, following which it was centrifuged at 7000 r.c.f. to pellet down the nuclei and cellular debris. The supernatant, which comprised the mitochondrial fraction was then aspirated. The pellet was further resuspended in 400 *μ*l of ice-cold buffer 3 (150 mM NaCl, 50 mM HEPES, 0.1% SDS, pH 7.4, with protease and phosphatase inhibitor cocktail) and vortexed intermittently for 30 min. The suspension was incubated at 4 °C in end-over-end rotation for 1 h and centrifuged at 7000 r.c.f. for 10 min at 4 °C. The supernatant, which contained the nuclei, was collected by aspiration.

### ATP determination

ATP was quantified using the ATP Determination Kit from Molecular Probes, Invitrogen (Carlsbad, CA, USA) following the manufacturer’s protocol. The assay was normalized with the amount of protein.

### LDH assay

LDH was spectrophotometrically measured in the cell lysates by the method of Bergmeyer^53^ at 37 °C by following the oxidation of NADH in 1 ml of medium consisting of 0.3 M mannitol, 10 mM KCl, 5 mM MgCl_2_, 10 mM KH_2_PO_4_ (pH 7.8) and 0.1% Triton X-100. The reaction was started by adding 50 mM pyruvate and the decrease in absorbance was monitored at 340 nm for 3 min. Activity was expressed as nmol NADH oxidized per min per mg protein (*ε*340=6220/M/l/cm).

### Cellular metabolites determination

Lactate concentration in the culture medium was measured using the Lactate Assay Kit (Abcam, Cambridge, UK). Citrate, pyruvate and acetyl coenzyme A were assayed using the commercially available kits from Abcam following the manufacturer’s protocol.

### MTT assay

Exponentially growing cells were seeded at 1×10^4^ cells per well in 96-well plates and incubated with rapamycin (100 nM)/torin1 (250 nM)/MK-2206 (10 *μ*M)/SB-415286 (30 *μ*M) for 24 h. MTT (5 mg/ml) was added to each well and the plates were incubated for 4 h at 37 °C. The formazan product was dissolved by adding 100 *μ*l DMSO to each well. The MTT absorbance value was detected at 590 nm in a microplate reader.

### Flow cytometric analysis

Detection of apoptotic cells was carried out using Annexin V/propidium iodide (PI) Staining Kit (Invitrogen). Cells were harvested and washed with cold PBS two times and resuspended in 200 *μ*l binding buffer at a concentration of 1×10^6^ cells per ml. The cells were incubated with 10 *μ*l Annexin V-fluorescein isothiocyanate and 5 *μ*l PI in the dark for 15 min. In total, 300 *μ*l binding buffer was then added in each tube before being analyzed with an LSRFortessa Cell Analyzer (BD Biosciences, San Jose, CA, USA).

### Quantitative PCR

Total RNA from cell homogenates was isolated using Trizol (Invitrogen) following the standard method and RNA quality was assessed by electrophoresis. To make cDNA, RT was performed using 1 *μ*g of total RNA and M-MLV reverse transcriptase (Ambion, Carlsbad, CA, USA). The RT conditions for each cDNA amplification were 95 °C for 5 min, 55 °C for 45 min and 70 °C for 15 min. The gene expression was quantified by SYBR green chemistry (FastStart Universal SYBR Green Master; Roche) in LightCycler 96 real-time PCR (Roche). Gene expression was normalized by 18S RNA expression by the ΔΔCt method.

### Oxygen consumption rate

HepG2 cells were plated at a density of 30 000 cells per well (XF24 cell culture microplate; Seahorse Biosciences, North Billerica, MA, USA). The cells were allowed to grow until confluent for 24 h, following which the cells were washed with XF Assay media (with 20 mM glucose, 1 mM sodium pyruvate and 1 mM glutamate, without sodium bicarbonate) and the cells were incubated for 1 h at 37 °C in a non-CO_2_ incubator. The sensor cartridge was allowed to hydrate in XF fluid 1 day before the experiment. The cartridge was allowed to calibrate in the Seahorse machine (North Billerica, MA, USA) for 20 min, following which the cell plate was allowed to run for the assay. The assay was normalized with protein and analyzed using the XFe 2.0.0 software (North Billerica, MA, USA).

### Cell proliferation/growth

HepG2 cells were plated at a density of 30 000 cells in 24-well culture plate and allowed to grow for 24 h. After 24 h, the cells were transfected with siPCK1 siRNA and kept for a further 12 h. Cells were treated with torin1 (250 nm) after 12 h of siRNA transfection and allowed to grow. After stipulated time periods of 12/24/48 h, cells were trypsinized and counted in a hemocytometer following the standard protocol.

### Pathway construction and expression analysis

The pathway relationships between mTOR and PCK1 and their selected regulators were collected from the KEGG pathway database^54^ and were represented as a metapathway network where edges represent activation/inhibition and boxes indicate proteins and/or complexes. mTOR-PCK1 subnetwork was created based on AKT-FOXO-based transcriptional regulation of PCK1, whereas other selected regulators of AKT, FOXO and PCK1 were also included in the network. Information regarding each step of gluconeogenesis pathway was collected from literature^55^ and was represented in cascade-pathway format.

The expression levels of genes involved in mTOR-PCK1 subnetwork (23 genes) and gluconeogenesis pathway (16 genes) were analyzed using gene expression data collected from the Oncomine database.^56^ Gene expression data from clinical specimens of cancer *versus* normal patient data sets were collected and differential expression analyses were performed using standardized normalization techniques and statistical calculations provided in the Oncomine website (https://www.oncomine.com/). Five data sets for HCC, 6 data sets for RCC and 6 data sets for CC were used for the differential expression analysis. Detailed information for all data sets is provided in Supplementary Table S1. Fold-change (FC) for a gene is defined as a change in the mRNA expression level of that gene in the cancer tissue compared with the normal expression level of the same gene for that tissue. Genes that are differentially expressing in ≥40% of the data sets with a log_2_ FC ≥1.5 and *P*-value ≤0.05 were considered as up/downregulated genes. FC and *P*-value for genes in all the data sets can be downloaded from www.hpppi.iicb.res.in/images/oncomine_pck1.zip.

### Statistics

Student’s paired two-tailed *t-*test was used to evaluate the statistical significance of the results. Pearson’s correlation coefficient ‘*r*’ was calculated to explore association between variables.

## Abbreviations

mTOR: mechanistic target of rapamycin
LDH: lactate dehydrogenase
PEPCK: phosphoenolpyruvate carboxykinase
OXPHOS: oxidative phosphorylation
SREBP: sterol regulatory element-binding protein
PTEN: phosphatase and tensin homolog
FAO: fatty acid oxidation
OCR: oxygen consumption rate
HCC: hepatocellular carcinoma
RCC: renal cell carcinoma
CC: colorectal carcinoma.
