# Supplementary material for: mTORC2 controls cancer cell survival by modulating gluconeogenesis
Source: Cell Death Discov. 2015 Sep 7;1:15016–. doi: 10.1038/cddiscovery.2015.16 (PMC4979518; doi:10.1038/cddiscovery.2015.16)
Supplement: Supplementary Data [file cddiscovery201516-s1.doc]

**SUPPLEMENTARY DATA**

**mTORC2 Controls Cancer Cell Survival by Modulating Gluconeogenesis**

**Md. Wasim Khan^1^, Dipsikha Biswas^1^, Mainak Ghosh^1^, Sapan Mandloi^2^**, **Saikat Chakrabarti^2^, Partha Chakrabarti ^1 *^**

^1^Division of Cell Biology and Physiology, CSIR-Indian Institute of Chemical Biology, Kolkata, India

^2^Division of Structural Biology and Bioinformatics, CSIR-Indian Institute of Chemical Biology, Kolkata, India

Table S1: Information of expression dataset that were used in this study.

| **Dataset** | **Cancer** | **Cancer Type** | **Number of cancer samples** | **Number of normal samples** | **Reference** |
| --- | --- | --- | --- | --- | --- |
| Chen liver | HCC | Hepatocellular Carcinoma | 104 | 76 | (1) |
| Mas liver | HCC | Hepatocellular Carcinoma | 38 | 19 | (2) |
| Roessler Liver | HCC | Hepatocellular Carcinoma | 22 | 21 | (3) |
| Roessler Liver 2 | HCC | Hepatocellular Carcinoma | 225 | 220 | (3) |
| Wurmbach Liver | HCC | Hepatocellular Carcinoma | 35 | 10 | (4) |
| Beroukhim Renal | RCC | Hereditary Clear Cell Renal Cell Carcinoma | 32 | 10 | (5) |
| Beroukhim Renal | RCC | Non-Hereditary Clear Cell Renal Cell Carcinoma | 27 | 10 | (5) |
| Gumz Renal | RCC | Clear Cell Renal Cell Carcinoma | 10 | 10 | (6) |
| Jones Renal | RCC | Clear Cell Renal Cell Carcinoma | 23 | 23 | (7) |
| Yusenko Renal | RCC | Clear Cell Renal Cell Carcinoma | 26 | 3 | (8) |
| Yusenko Renal | RCC | Clear Cell Renal Cell Carcinoma | 19 | 3 | (8) |
| Hong Colorectal | CC | Colorectal Carcinoma | 70 | 12 | (9) |
| Kaiser Colon | CC | Colon Adenocarcinoma | 41 | 5 | (10) |
| Sabates-Bellver Colon | CC | Colon Adenoma | 25 | 15 | (11) |
| Skrzypczak Colorectal | CC | Colorectal Adenocarcinoma | 45 | 24 | (12) |
| Skrzypczak Colorectal | CC | Colorectal Carcinoma | 36 | 24 | (12) |
| TCGA Colorectal | CC | Colon Adenocarcinoma | 102 | 19 | (13) |
|  |  |  |  |  |  |

References for Table S1

1. Chen X, Cheung ST, So S, Fan ST, Barry C, Higgins J et al. Gene expression patterns in human liver cancers. *Mol. Biol. of the Cell* 2002; **13:** 1929-1939
2. Mas VR, Maluf DG, Archer KJ, Yanek K, Kong X, Kulik L et al. Genes involved in viral carcinogenesis and tumor initiation in hepatitis C virus-induced hepatocellular carcinoma. *Mol. Med.* 2009; **15:** 85- 94
3. Roessler S, Jia HL, Budhu A, Forgues M, Ye QH, Lee JS et al. A unique metastasis gene signature enables prediction of tumor relapse in early-stage hepatocellular carcinoma patients. *Cancer Res.* 2010; **70:** 10202- 10212
4. Wurmbach E, Chen YB, Khitrov G, Zhang W, Roayaie S, Schwartz M et al. Genome-wide molecular profiles of HCV-induced dysplasia and hepatocellular carcinoma. *Hepatology* 2007; **45:** 938- 947
5. Beroukhim R, Brunet JP, Di Napoli, A, Mertz KD, Seeley A, Pires MM et al. Patterns of gene expression and copy-number alterations in von-hippellindau disease-associated and sporadic clear cell carcinoma of the kidney. *Cancer Res* 2009; **69:** 4674- 4681
6. Gumz ML, Zou H, Kreinest PA, Childs AC, Belmonte LS, LeGrand SN et al. Secreted frizzled-related protein 1 loss contributes to tumor phenotype of clear cell renal cell carcinoma. *Clinical Cancer Res.* 2009; **13:** 4740- 4749
7. Jones J, Otu H, Spentzos D, Kolia S, Inan M, Beecken WD et al. Gene signatures of progression and metastasis in renal cell cancer *Clinical Cancer Res.* 2005; **11:** 5730- 5739
8. Yusenko MV, Kuiper RP, Boethe T, Ljungberg B, van Kessel AG and Kovacs G. High-resolution DNA copy number and gene expression analyses distinguish chromophobe renal cell carcinomas and renal oncocytomas. *BMC Cancer* 2009; ***9*:** 152
9. Hong Y, Downey T, Eu KW, Koh PK and Cheah PY. A 'metastasis-prone' signature for early-stage mismatch-repair proficient sporadic colorectal cancer patients and its implications for possible therapeutics. *Clin. Exp. Metastasis* 2010; ***27:*** 83- 90
10. Kaiser S, Park YK, Franklin JL, Halberg RB, Yu M, Jessen WJ, Freudenberg J et al. Transcriptional recapitulation and subversion of embryonic colon development by mouse colon tumor models and human colon cancer. *Genome Boil.* 2007; **8:** R131
11. Sabates-Bellver J, Van der Flier LG, de Palo M, Cattaneo E, Maake C, Rehrauer H et al. Transcriptome profile of human colorectal adenomas. *Mol. Cancer Res.* 2007; ***5*:** 1263- 1275
12. Skrzypczak M, Goryca K, Rubel T, Paziewska A, Mikula M, Jarosz D et al. Modeling oncogenic signaling in colon tumors by multidirectional analyses of microarray data directed for maximization of analytical reliability. *PloS One* 2010; *5*.
13. Comprehensive molecular characterization of human colon and rectal cancer. *Nature* 2012; **487:** 330-337

**Figure S1. Effects of mTOR inhibition in Huh7 cells**. A. HuH7 cells were incubated with 100nM rapamycin (Rapa), 250nM of torin 1 (Tor) and vehicle (Con) for 24 h and whole cell lysates wereanalyzed by immunoblotting with indicated antibodies. β Actin serves as loading control. B. HuH7 cells were treated with Rapa or Tor followed by incubation with^14^C labelled acetate for 2h. The incorporation of ^14^C in the lipid phase was measured in triplicatesamples in a beta scintillation counter and normalized with total cellular proteins. C. Glucose consumption was measured in HuH7 cells after Rapa/Tor treatment for 24 h. D. Glucose uptake was quantified in HuH7 cells after treatment with Rapa/Tor for 24 hrs. E. Following treatment with Rapa and Tor lactate was measured in the media. All panels: ^*^p < 0.05, ^**^p < 0.01, ^***^ p < 0.001 compared to Con cells.


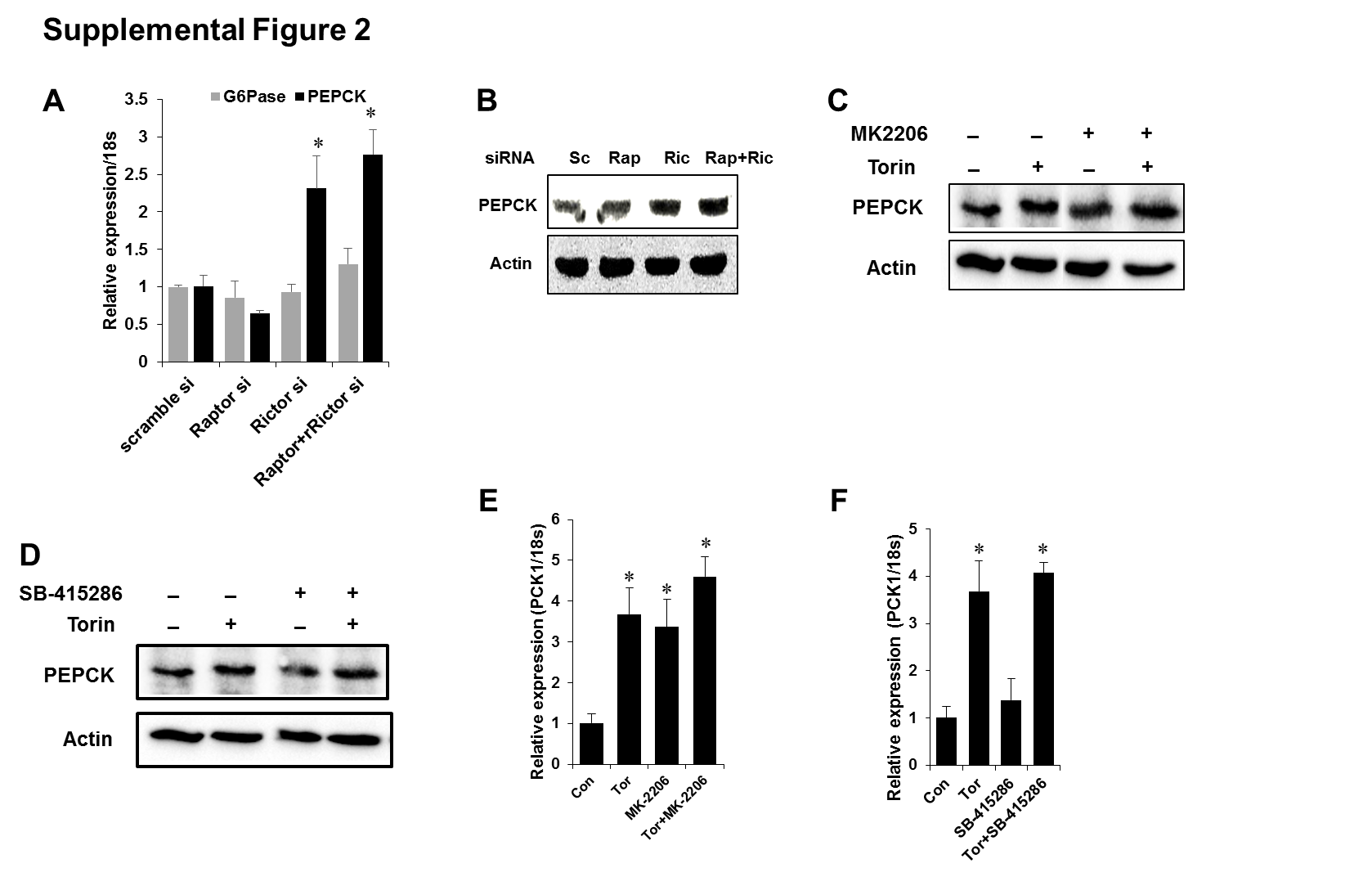


**
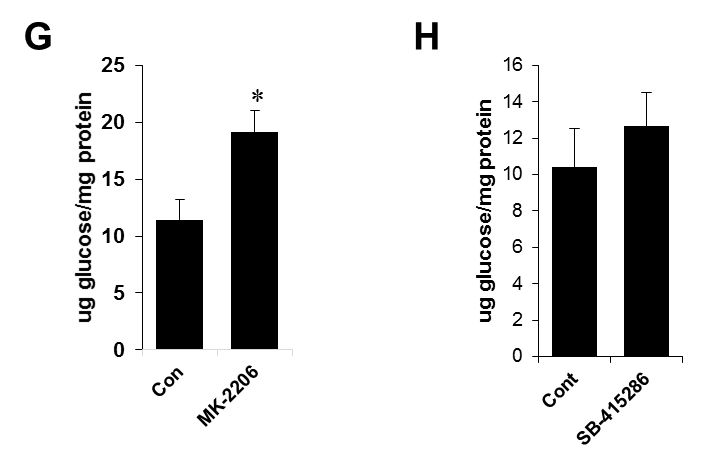
**

**Figure S2. Regulation of Gluconeogenesis following mTOR, Akt and GSK3 inhibition in HepG2 cells.** A. HepG2 cells were transfected with scrambled siRNA (sc) and siRNA against raptor (Rap), rictor (Ric) or both (Rap+Ric) and mRNA expression of *G6PC* and *PCK1* were measured by quantitative PCR in triplicate samples. B-D whole cell lysates were analyzed for PEPCK1 expression by Immunoblotting after treatment with Rapamycin/Torin/MK-2206/SB-415286. E-F. HepG2 cells were treated with Torin 1/MK-2206/Sb-415286 for 24 h and the PCK1 levels were quantified by quantitative PCR. G-H. Glucose production was quantified in HepG2 cells after treatment with MK-2206 or SB-415286 as described in Methods. All panels: ^*^p < 0.05, ^**^p < 0.01, compared to sc or Con cells


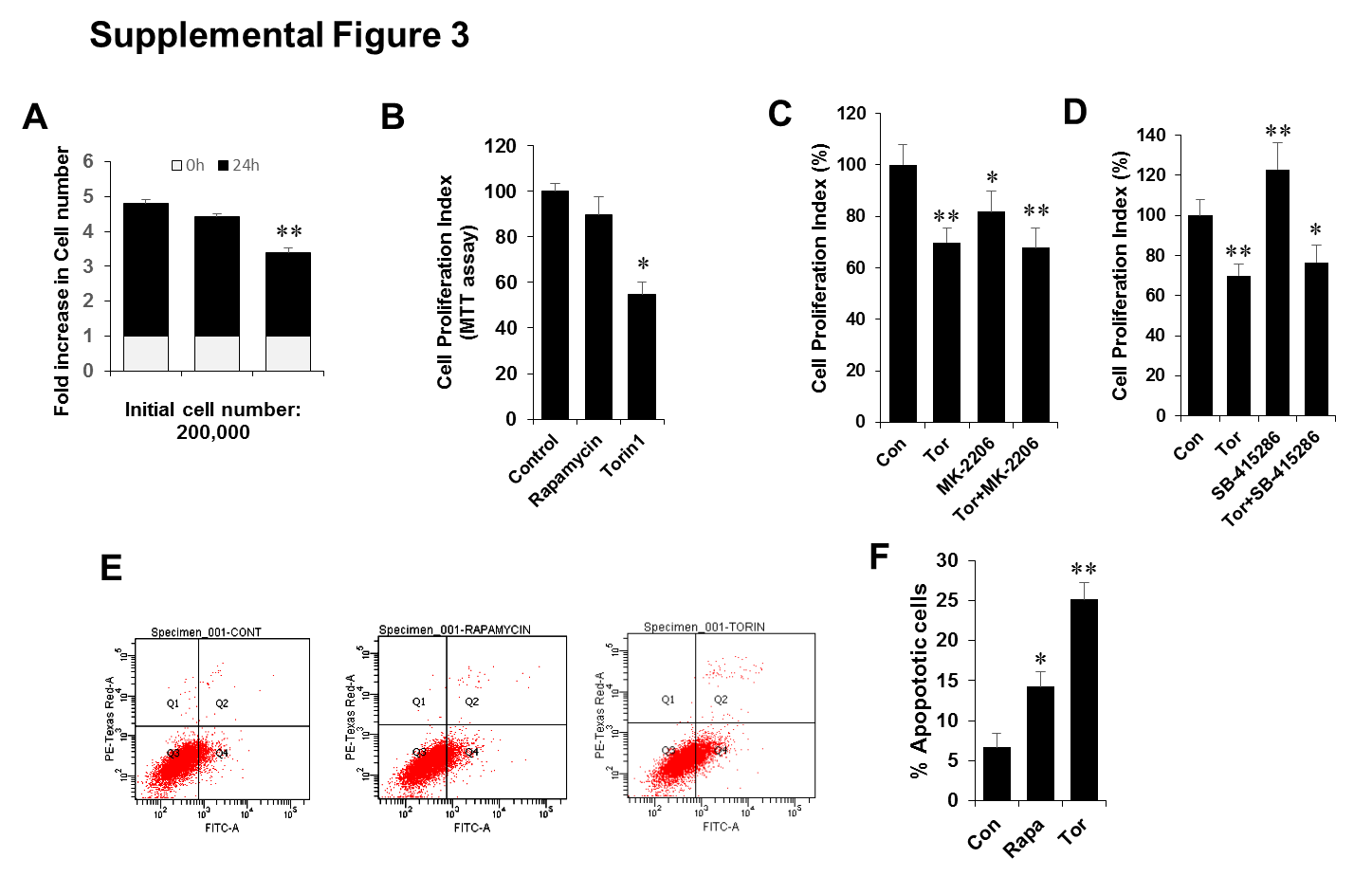


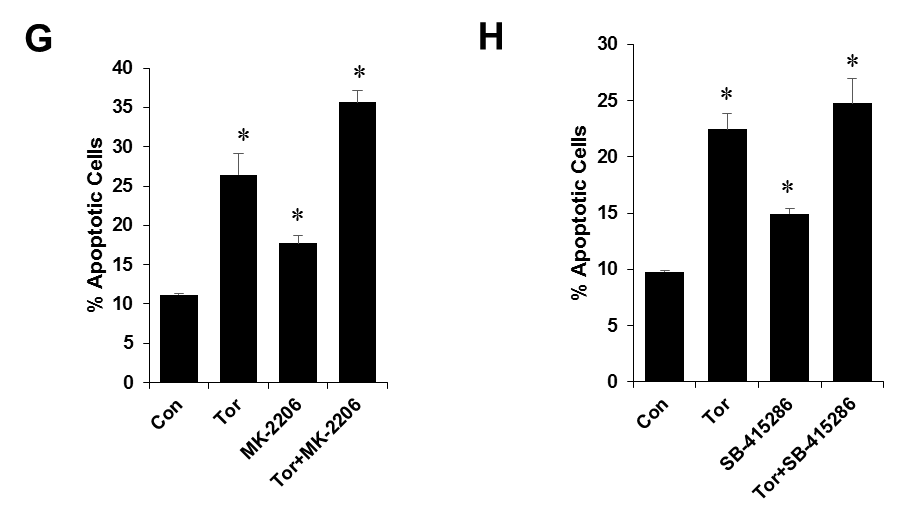


**Figure S3. Effects on cell proliferation and survival upon mTOR, Akt and GSK3 inhibition.** A. Cell number was counted in a hemocytometer after Rapa/Tor treatment as described in Methods. B-D. MTT assay was performed in HepG2 cells after 24 h treatment with Rapamycin/Torin/MK-2206/SB-415286. E-H. Cells undergoing apoptosis were quantified by Annexin V binding as described in Methods. Graphical representation of data from C. All panels: ^*^p < 0.05, ^**^p < 0.01, compared to Con cells


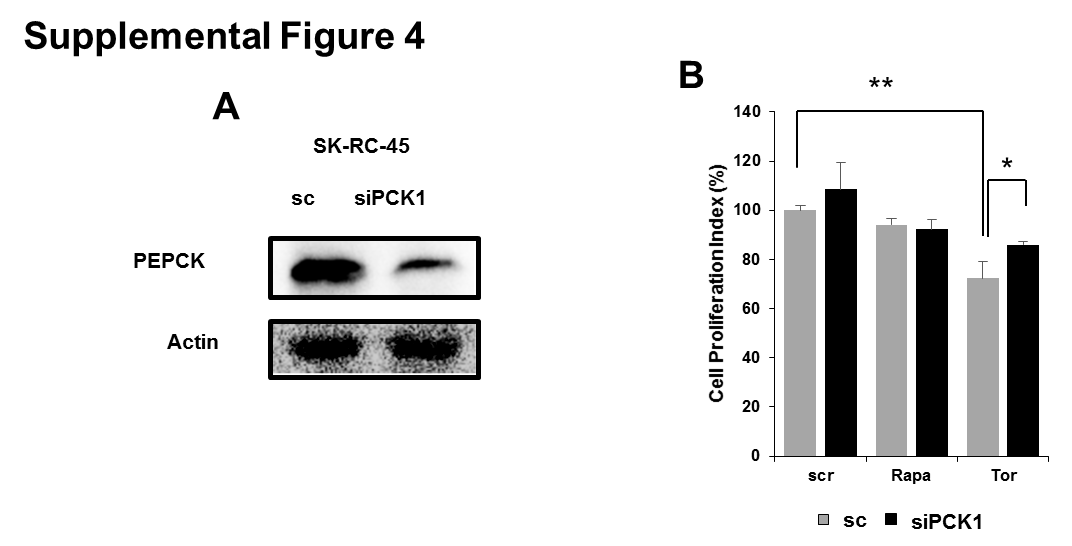


**Figure S4. Effects on cell proliferation upon PCK1 knockdown in RCC cells.**A. SK-RC-45 cells were transfected with scrambled siRNA (sc) and siRNA against *PCK1*. Whole cell lysates were analyzed for PEPCK protein expression. B. Cell proliferation using MTT assay after si*PCK1* knockdown followed by Rapa and Tor treatment. All panels: ^*^p < 0.05, ^**^p < 0.01 compared to Con cells.


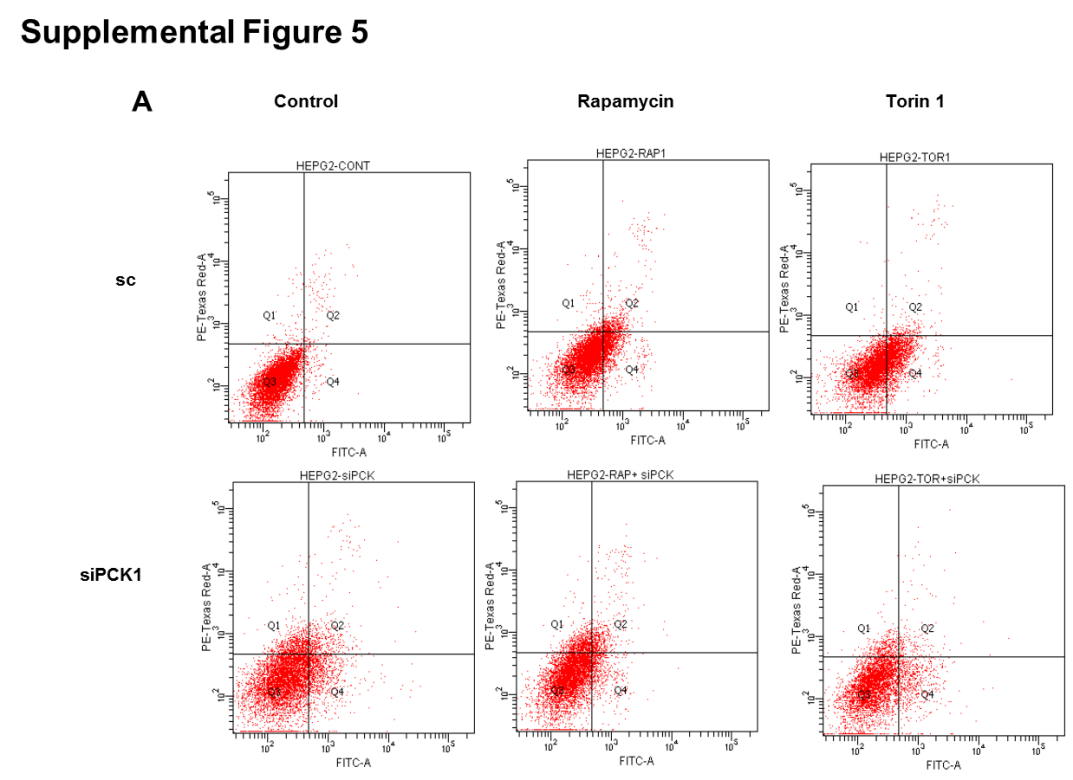


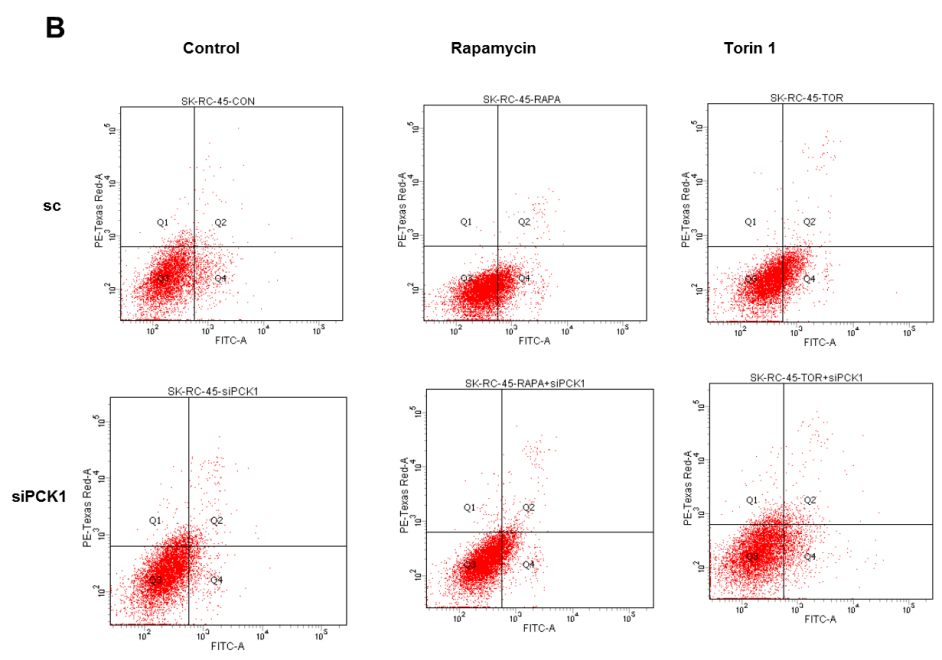


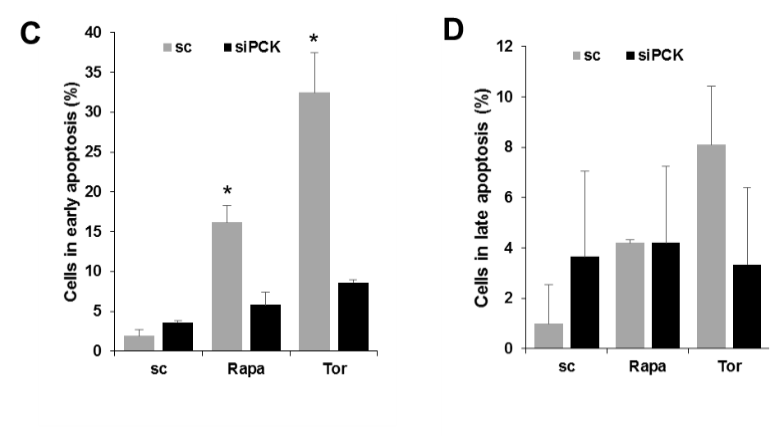


**Figure S5: Cell survival following PCK1 knockdown.** A. HepG2 cells were transfected with siRNA against PCK1 and following 24 h incubation the cells were treated with Rapa or Tor and subjected to flow cytometry analysis for apoptosis detection as described in Methods. B. SK-RC-45 cells were transfected with siRNA against PCK1 and following 24 h incubation the cells were treated with Rapa or Tor and subjected to flow cytometry analysis for apoptosis detection as described in Methods. C-D. Graphical representation of data from Supplemental Fig 5 B. All panels: ^*^p < 0.05, ^**^p < 0.01 compared to Con or sc cells.

**Figure S6. Effects on cell proliferation upon G6Paseα knockdown in HepG2 cells.** A. HepG2 cells were transfected with scrambled siRNA (sc) and siRNA against *G6Paseα* and mRNA levels of *G6Paseα* were analyzed by quantitative PCR and normalized to 18S RNA expression. B. After 24 h post-transfection with scrambled (sc) and si*G6Paseα*, cells were treated with Tor for another 24 h as indicated and glucose production was measured in media. C. Cell proliferation using MTT assay after si*G6Paseα* knockdown followed by Tor treatment. D. Percentage of apoptotic cells were determined after treatment siRNA transfection and treatment with Torin 1 (250nm) by flow cytometry. All panels: ^*^p < 0.05, ^**^p < 0.01 compared to Con cells.
